# Comparative Evaluation of Distortion in Wax Patterns Fabricated Using Conventional and Electrical Heat Sources: An In Vitro Study

**DOI:** 10.7759/cureus.41235

**Published:** 2023-06-30

**Authors:** BarathSundar A, Saravanan M, Muthukumar B

**Affiliations:** 1 Prosthodontics, SRM Dental College, Chennai, IND

**Keywords:** distortion, linear shrinkage, dimensional stability, marginal accuracy, wax pattern fabrication, inlay waxes, dental restoration

## Abstract

Introduction

Tooth loss significantly impacts individuals’ functional capabilities and quality of life. Fixed partial dentures have been a reliable treatment method for tooth replacement, with their fabrication often involving waxes. Waxes play a crucial role in creating a wax pattern in dental restoration; in particular, inlay waxes play a role in the shape, size, and contour of the restorations. However, these waxes have inherent disadvantages, including a high thermal expansion coefficient and propensity to warp or distort over time. This study aimed to compare wax patterns derived from two heat sources, an electric heat source and a conventional flame, to enhance their marginal accuracy and dimensional stability.

Methods

This study used an abutment resembling a prepared maxillary right central incisor designed via computer-aided design software and milled from zirconia. Inlay wax was melted using either an electrically heated spatula or a conventional flame, poured into a metal sleeve or a cuboidal mold, and allowed to cool to room temperature. The wax patterns were stored at room temperature for one hour and 24 hours. Subsequently, linear and volumetric measurements were taken to assess the shrinkage of the wax patterns.

Results

Patterns fabricated using the electric heat source showed less shrinkage at both time points for linear shrinkage and at one hour for volumetric shrinkage than those made using the conventional flame. However, by the 24th hour, patterns made with the electric heat source showed more volumetric shrinkage than those made with the conventional heat source. Significant shrinkage was observed between one hour and 24 hours for both heat sources, suggesting that wax patterns should be invested immediately after fabrication for maximum precision.

Conclusions

The results suggest that electric heating may be a viable alternative to conventional flame for minimizing discrepancies in wax patterns, particularly in the initial stages of wax pattern fabrication. However, prolonged use may lead to greater volumetric shrinkage with electric heating. These findings point to the potential of electric heating as an alternative to conventional flame in dental restorations, although further research is needed to validate and expand upon these findings.

## Introduction

Tooth loss and the loss of related structures can significantly affect functional capabilities and potentially decrease the quality of life of those afflicted [1]. For several decades, fixed partial dentures have been a reliable treatment method for replacing missing teeth [2]. Fabricating these dentures typically involves using various types of waxes, which have wide-ranging applications in dentistry, from rudimentary tasks to more precision-oriented work. This includes inlay wax, casting wax, and baseplate wax [3,4].

In dentistry, waxes are crucial for creating a wax pattern, typically handled by inlay casting waxes [5]. These waxes, a mixture of natural and synthetic varieties, come in two types: Type 1 medium wax for direct techniques and Type 2 soft wax for indirect techniques. Inlay wax helps shape the restoration’s predefined size and contour. Once the pattern is set, it undergoes an investment process, where it is converted to a more durable material like cast alloys and then layered with ceramics. As per the American National Standards Institute/American Dental Association specification No. 4, the maximum permitted flow for Type 1 waxes at 37 °C is 1%, whereas both Type 1 and Type 2 waxes at 45 °C should maintain a minimum flow of 70% and a maximum flow of 90% [6,7]. Notably, inlay waxes exhibit a high thermal expansion coefficient. For example, a temperature increase of 20 °C results in a linear expansion of 0.7%, while cooling from 37 °C to 25 °C can lead to a contraction of up to 0.35%. This variability is due to wax having melting ranges rather than fixed melting points, owing to the presence of molecularly similar components with different molecular weights.

Metal-ceramic restorations, which include artificial crowns or fixed complete or partial dentures with a metal substructure and porcelain veneer, are a crucial component of dentistry [8]. As a versatile material in the fabrication of fixed dental prostheses, metal-ceramic is often the go-to choice for tooth replacements [9,10]. During the creation of cast restorations, the “lost wax” technique is typically favored for its accuracy and attention to detail [11,12]. The precision of the wax pattern greatly influences the fit and intricacy of the restoration, and inlay waxes, with their ease of manipulation and superior detail reproduction, are widely employed for dental castings.

Despite their benefits, waxes have inherent disadvantages, including a high thermal expansion coefficient and a propensity to warp or distort over time. During fabrication, wax patterns can develop internal strains regardless of the manipulation method, leading to distortion when the strain relaxes. Both time and temperature can exacerbate wax distortion; thus, it is generally advised that wax patterns be invested immediately after removal from the die or cast.

Given that wax patterns substantially affect the final restoration’s fit and detail and their high thermal expansion coefficient, the heat sources used for wax pattern manipulation may also influence the pattern’s accuracy. This study aims to compare patterns derived from two heat sources to enhance wax patterns’ marginal accuracy and dimensional stability.

## Materials and methods

Linear shrinkage evaluation: preparation of test sample

**Figure 1:**
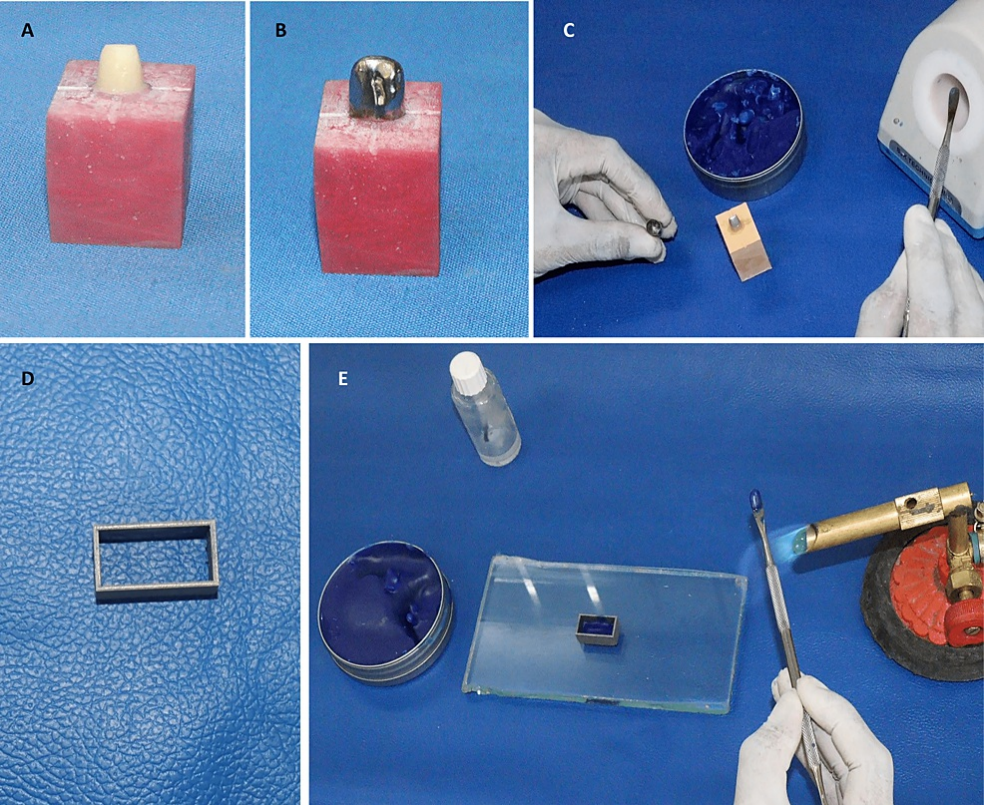
(A) Master die of maxillary central incisor; (B) custom sleeve; (C) fabrication of samples for linear shrinkage using an electrical heat source; (D) master die for volumetric shrinkage measurement; and (E) fabrication of samples for volumetric shrinkage using a conventional heat source

An abutment was designed resembling a prepared maxillary right central incisor using exocad GmbH computer-assisted design (CAD) software (Align Technology, Inc., Darmstadt, Germany). The abutment featured a shoulder finish line with dimensions of 6 mm faciolingually and 7 mm mesiodistally near the finish line, a facial height of 7 mm, mesial and distal heights of 6 mm, a palatal height of 4 mm, and a uniform taper of 12°. Milling of this designed abutment from zirconia was done to create the master die (Figure 1A), which was secured onto a vertically placed 3.5-mm platform implant analog (Adin Dental Implant System Ltd., Afula, Israel). This analog was embedded in an auto-polymerizing poly-methyl methacrylate resin block (DPI RR Cold Cure, Dental Products of India, Mumbai, India) measuring 35 mm in length, 25 mm in width, and 10 mm in height. Four standard reference points were established (mid-facial, mid-lingual, mid-mesial, and mid-distal) on the acrylic block adjacent to the milled zirconia abutment, each separated by a 90° angle. These reference points were essential for measuring the vertical marginal discrepancy of the wax patterns [13].

Designing the custom sleeve without undercuts was done over the master die (Figure 1B) using exocad GmbH CAD software (Align Technology, Inc.). This sleeve was fabricated using direct metal laser sintering and served as the standard sleeve for wax pattern fabrication. After fabricating a custom tray over the master die with auto-polymerizing poly-methyl methacrylate resin (DPI RR Cold Cure), an impression was made of the master die using polyvinyl siloxane elastomeric impression material (Express, 3M ESPE, St. Paul, Minnesota, USA). Die stone was mixed (Ultrarock, Kalabhai, Mumbai, India) using a vacuum mixer, poured the impressions, and fabricated 44 individual die stone molds. All dies received two coats of die spacer (Giroform Die Link, AmannGirrbach, Germany), applied 1 mm short of the margins.

Group A

Inlay wax (Bego Crown Wax, Bego Bremer, Germany) was melted using an electrically heated spatula (Ultra-Waxer 2, Kerr Dental, Brea, California, USA) at a temperature range of 36 °C to 260 °C (Figure 1C). After lubricating the stone dies and the metal sleeve, poured the molten wax into the metal sleeve, fastened it to the stone die, and allowed it to cool to room temperature. Excess wax was then carved away, made any necessary margin carvings, and separated the patterns from the gypsum dies. Wax patterns were then stored at room temperature for one hour and 24 hours before reseating them on their respective dies using gentle finger pressure. Measuring the marginal gaps between the predetermined points on the die and the margin of the wax pattern using a vision measuring machine (OPUS 3D, Bruker, Billerica, Massachusetts, USA) was done at both the first and 24th hour, calculating an average from the four values (midfacial, mid lingual, mid mesial, and mid distal) obtained for statistical analysis.

Group B

The same process was followed as in Group A, but melting the inlay wax (Bego Crown Wax, Bego Bremer, Germany) was done with a No. 7 P.K. Thomas spatula (GDC Dental, London, Essex, UK) over the blue flame of a Bunsen burner.

Volumetric shrinkage evaluation: preparation of test samples

Designing a cuboidal mold with dimensions of 20 mm in length, 10 mm in width, 10 mm in height, and a thickness of 5 mm was done using Fusion 360 software (Autodesk Inc., San Francisco, California, USA). Then milling this design in metal was done (Figure 1D). This cuboidal mold was a template for creating samples to assess volumetric shrinkage.

Group A

Inlay wax (Bego Crown Wax, Bego Bremer, Germany) was melted using an electrically heated spatula (Ultra-Waxer 2, Kerr, Kloten Switzerland) from 36 °C to 260 °C. After lubricating the cuboidal metal mold, the heated wax was poured into it and allowed to cool to room temperature. Then the excess wax was carved away, if necessary. The patterns were then separated from the mold and stored at room temperature for intervals of one hour and 24 hours. Subsequently, each sample’s dimensions (length, breadth, height) were measured using a vision measuring machine (OPUS 3D) at the first and 24th hour. The volume of each sample was calculated using the formula length × breadth × height, and the values obtained were used for statistical analysis.

Group B

The same process as in Group A was followed, but melting the inlay wax (Bego Crown Wax, Bego Bremer, Germany) was done with a No. 7 P.K. Thomas spatula (GDC Dental) over the blue flame of a Bunsen burner (Figure 1E).

## Results

Table 1 presents the mean values for Group A’s linear shrinkage. After one hour and 24 hours, the conventional heat source group’s mean values were higher than those of the electric heat source, with respective values of 0.38 and 0.84, which suggests that the linear shrinkage was greater with the conventional heat source. After the first hour, the standard deviations for the conventional and electric heat sources were 0.01 and 0.02, respectively. After 24 hours, these values were 0.03 and 0.04.

**Table 1:** Linear Shrinkage by heat technique SD, standard deviation; SE, standard error

Time	Heat Technique	N	Mean	SD	SE
1 hour	Conventional	22	0.38	0.01	0.003
	Electric	22	0.30	0.02	0.003
24 hours	Conventional	22	0.84	0.03	0.005
	Electric	22	0.78	0.04	0.009

Table 2 presents the mean values for Group B’s volumetric shrinkage. The mean values for the conventional and electric heat sources after one hour were 1926.52 and 1945.43, respectively, whereas, after 24 hours, they were 1603.91 and 1551.18. This suggests that the conventional heat source’s volumetric shrinkage was greater after one hour, but after 24 hours, the electric heat source’s shrinkage was greater. After one hour, the standard deviations for the conventional and electric heat sources were 41.71 and 19.65, respectively, with corresponding standard errors of 8.89 and 4.19. After 24 hours, the standard deviations were 106.18 and 210.14, with standard errors of 22.64 and 44.80.

**Table 2:** Volumetric shrinkage by heat technique SD, standard deviation; SE, standard error

Time	Volumetric	N	Mean	SD	SE
1 hour	Conventional	22	1926.52	41.71	8.89
	Electric	22	1945.43	19.65	4.19
24 hours	Conventional	22	1603.91	106.18	22.64
	Electric	22	1551.18	210.14	44.80

## Discussion

Inlay wax is primarily used for fabricating cast restorations using the “lost wax” technique. The fit and fine details of the wax pattern greatly influence the accuracy of these restorations, making inlay waxes popular for dental castings due to their good detail reproduction and ease of manipulation.

Storage temperature, storage time, and wax manipulation temperature can affect wax pattern distortion. Phillips et al. found that distortion in casting was not routinely observed until 45 minutes of storage, with distortion worsening as storage time increased [14]. Significant distortion was observed when patterns were stored for 12 and 24 hours, with patterns stored at 36 °C or 96.8 °F showing significantly less distortion than those stored at 115 °F. Ito et al. explored minimizing wax distortion by altering its composition [15].

Since the fit and detail of a cast restoration depend greatly on the accuracy of the wax pattern, which has a high coefficient of thermal expansion, the heat sources used for manipulating the wax pattern may affect its accuracy. Our study evaluated the linear and volumetric shrinkage of wax patterns fabricated using a conventional Bunsen burner heat source and an electrical heat source at two time intervals: one hour and 24 hours. The results showed that patterns fabricated using the electric heat source had less shrinkage at both time points for linear shrinkage and one hour for volumetric shrinkage. However, by the 24th hour, patterns made with the electric heat source showed more volumetric shrinkage than those from the conventional heat source.

We also observed significant shrinkage between the first and 24 hours, suggesting that wax patterns should be invested immediately after fabrication for maximum precision. Storage of wax patterns over 24 hours led to significant distortion, making them inferior to the required dental accuracy. However, both heat sources produced patterns within the dental accuracy range at the first hour, with those from the electric heat source slightly superior. This superiority may be due to the electric heat source’s efficiency, fast heating, cleanliness, safety [16], and absence of carbon residues commonly found in a conventional Bunsen burner flame [17]. 

Previous studies [18,19] have shown that the most common injuries in an institutional setup were due to burns from Bunsen burner flame. Hence, instead of a conventional Bunsen burner, which has naked flame, we can opt for an electrical heat source, which is safer. Additionally, the uniform and constant heat from the electric heat source could contribute to the superior quality of patterns fabricated via this method [20].

Also, since electric heating has been proven to significantly reduce CO_2_ emissions and improve atmospheric quality [21], as well as the advancements in electric heating technology leading to carbon emission mitigation in China [22], the benefits of electric heat over conventional Bunsen burner flames are evident. Conventional flames often lack uniformity and result in higher concentrations of unburnt carbon when exposed to aerial oxygen [23].

Our study had several important limitations. First, the study was confined to two methods of heating-conventional flame and electric heating-and did not consider other possible heat sources. Furthermore, the influence of environmental factors such as humidity and temperature, which can potentially affect wax pattern distortion, was not controlled or accounted for. Additionally, our sample size was limited, which may affect the generalizability of the results. Finally, this research was restricted to in vitro conditions, which might not accurately mimic the in vivo conditions in clinical practice. As such, further research that accounts for these limitations is necessary to validate and expand upon our findings.

## Conclusions

In light of the limitations of this study, we can draw several conclusions. Firstly, the volumetric shrinkage in the wax patterns fabricated using the electric heat source was less than that created with the conventional flame during the first hour. However, after 24 hours, the situation reversed, with the patterns from the electric heat source experiencing higher volumetric shrinkage. Secondly, regardless of the time frame, the electric heat source consistently resulted in less linear shrinkage in the wax patterns than that created using the conventional flame. Finally, wax patterns fabricated using both heat sources during the first hour remained within the acceptable range for dental accuracy. These findings suggest that electric heating may be a viable alternative to the conventional Bunsen burner flame for minimizing discrepancies in wax patterns. It seems plausible that electric heating could replace the Bunsen burner flame in this context.
